# Applying Linear Mixed Effects Models (LMMs) in Within-Participant Designs With Subjective Trial-Based Assessments of Awareness—a Caveat

**DOI:** 10.3389/fpsyg.2018.00788

**Published:** 2018-05-25

**Authors:** Guido Hesselmann

**Affiliations:** Visual Perception Laboratory, Department of Psychiatry and Psychotherapy, Charité—Universitätsmedizin Berlin, Corporate Member of Freie Universität Berlin, Humboldt-Universität zu Berlin, Berlin Institute of Health, Berlin, Germany

**Keywords:** *post hoc* trial sorting, anova, linear mixed models, unbalanced data, visibility, consciousness, awareness

## Introduction

In experimental psychology, including consciousness research, within-participant designs are typically more powerful than between-participant designs. During the last decades or so, the most widely used statistical method to analyze data from within-participant designs has been the repeated-measures (rm-) ANOVA. In recent years, however, empirical studies have increasingly turned toward using linear mixed effects models (LMM) to analyze data from within-participant designs (Baayen et al., 2008; Magezi, 2015).

LMMs are sometimes preferred over rm-ANOVA for a single practical reason, namely their ability to deal with unbalanced and incomplete data sets. For the sake of illustration, I will briefly describe a simple psychophysical experiment. Participants are instructed to respond as quickly and as accurately as possible to some predefined feature of a visual target (e.g., its semantic category). Response times (RTs) are recorded as the dependent variable. Immediately before the presentation of the target, another visual stimulus is presented. In the following, I will refer to this stimulus as the cue stimulus. The cue stimulus is presented at the threshold (liminally) or near the threshold of perception (peri-liminally), so that participants sometimes see the stimulus, and sometimes miss it. Liminal presentation can be achieved by low visual contrast, brief presentation times, and various psychophysical suppression methods (Breitmeyer, 2015). On each trial, participants first provide the speeded response to the target stimulus, and then rate the subjective visibility of the liminal cue stimulus using a binary scale (“seen,” “not seen”). Alternatively, a continuous subjective visibility scale such as the perceptual awareness scale (PAS) can be binarized by selecting and/or pooling across ratings.

A classical research question would be whether some features of the cue facilitate the response to the target (i.e., priming). Based on the subjective trial-based assessment of awareness, or *post hoc* trial sorting, priming can be compared when the cue is not consciously perceived relative to when it is (Van den Bussche et al., 2013; Avneon and Lamy, 2018). Another research question could be whether the visibility of the cue stimulus itself affects the RTs to the target stimulus. Due to cognitive costs associated with seeing the cue, responses to targets preceded by “seen” cue stimuli may be slower or less accurate than the responses to targets preceded by “unseen” cue stimuli.

In these examples, one commonly used statistical approach is to first sort trials into “seen” and “not seen” trials, and then average and submit the data to rm-ANOVA^1^.

In the absence of individual adjustment of stimulation parameters, it is a realistic scenario that the distribution of visibility ratings will be different for individual participants. In the extreme case, some participants might only rarely, or even never provide “seen” ratings, while other participants mostly provide “seen” ratings and therefore hardly any or no “not seen” ratings. Since the rm-ANOVA is based on the mean RTs per condition, participants with no “seen” trials or no “not seen” trials will be removed from the statistical analysis. Some researchers might also be reluctant to use mean RTs based on < 10 trials or so. In this situation, and primarily for the pragmatic reason to use most of the obtained experimental data, the researcher might decide to calculate LMMs, which can accommodate missing data. Before introducing the data simulation, the next paragraph provides a brief overview of the assumptions for rm-ANOVA and LMMs.

## Assumptions of rm-ANOVA and LMMs

Rm-ANOVA and LMMs are extensions of linear regression, and a number of assumptions are therefore common to both methods (Field, 2012). Among these, the absence of correlation with external variables is most important for the current data report. Beyond the common assumptions, rm-ANOVA additionally requires compound symmetry and complete data (Magezi, 2015). Compound symmetry means that the variances as well as the covariances of the repeated measures are similar (or, homogeneous). If the stringent assumption of compound symmetry is violated, then the sphericity assumption is still a necessary and sufficient condition for the F*-*tests to be valid. Sphericity means that the variances of the difference scores (between the levels of the repeated factor) are similar. The assumption of sphericity is often violated in experimental psychology, which may increase Type I errors, but this violation can be accounted for by correcting the degrees of freedom (e.g., using the Greenhouse-Geisser correction). Please note that in the current data simulation, the sphericity assumption is met because the repeated factor has only two levels (“seen” and “not seen”; see data simulation below). Follow-up simulations employing graded levels of visibility will need to consider sphericity when calculating rm-ANOVA. Complete data, the second additional assumption of rm-ANOVA, means that, for each participant, measurements must be available for all levels of the repeated factor. In contrast to rm-ANOVA, LMMs do not depend on assumptions about the variance-covariance matrix, and LMMs can accommodate missing data (Magezi, 2015).

## Simulated data sets (methods)

In this data report, unbalanced within-participant data sets were generated in a numerical simulation, in order to compare the statistical outcomes of rm-ANOVA and LMMs. The R code for the data simulation is available at the Center for Open Science (OSF; https://osf.io/d7y8h/).

**Figure 1:**
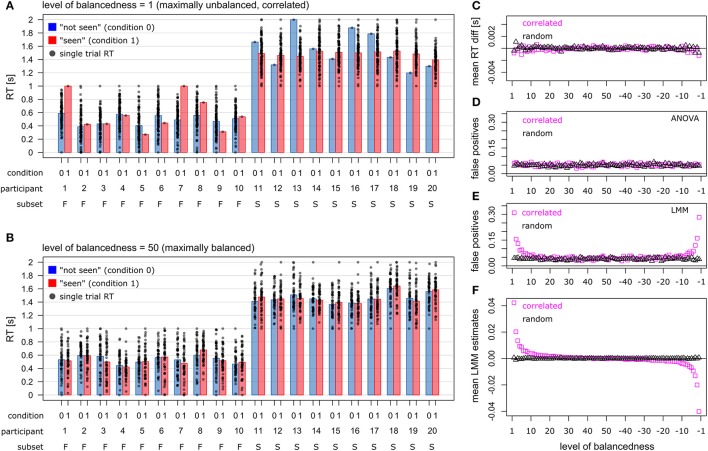
Simulated data sets and statistical outcomes. **(A)** Maximally unbalanced data set (level 1). Participants 1–10 produced 1 trial in condition 1 (“seen”, red bars), and 99 trials in condition 0 (“not seen,” blue bars). Participants 11–20 showed the inverse pattern. Simulated is a tight relationship between the visibility ratings and RTs, such that participants 1–10 had shorter RTs than participants 11–20 (correlated case). **(B)** Fully balanced data set (level 50). Each participant produced 50 trials in condition 0, and 50 trials in condition 1. **(C)** Mean RT differences between the two visibility conditions, across all levels of balancedness. **(D)** False positive rate for rm-ANOVA. **(E)** False positive rate for LMM. **(F)** Mean LMM estimates for fixed effect “visibility” across all levels of balancedness. **(C–F)** magenta squares indicate the correlated case, and black triangles indicate the random case.

In total, 100 trials per participant (*N* = 50) were generated. The level of balancedness ranged from 1 to 50. At level 1, one trial was rated as “seen,” while 99 trials were rated as “not seen” (Figure 1A). At level 50, 50 trials were rated as “seen,” and 50 trials were rated as “not seen,” thus indicating maximal balancedness (Figure 1B). Negative levels of balancedness indicate the inverse pattern, such that at level −1 one trial was rated as “not seen,” while 99 trials were rated as “seen.” Accordingly, levels 1 and −1 indicate the lowest level of balancedness.

Figures 1A,B illustrates that two subsets of participants were simulated: participants with short RTs (subset “Fast”, F), and participants with long RTs (subset “Slow,” S). Subset “Fast” (*N* = 25) had RTs ranging from 0s to 1s (mean 0.5s), while subset “Slow” (*N* = 25) had RTs ranging from 1s to 2s (mean 1.5s). Both RT distributions were based on the *rnorm* function in R (mean = 1, standard deviation = 1), and rescaled to the range 0–1s. For subset “Slow”, 1s was added to each RT data point. Note that for the sake of illustration, Figures 1A,B plots the data from only 20 participants (subset “Fast”: 1–10, subset “Slow”: 11–20).

Two different relationships between the participants' subset membership and the pattern of subjective visibility ratings were simulated. In the “correlated” case, a tight relationship was simulated (Figure 1A). For example, at balancedness level 1 in subset “Fast,” one trial was rated as “seen,” and 99 trials were rated as “not seen.” Subset “Slow” showed the inverse rating pattern so that at level 1 one trial was rated as “not seen,” while 99 trials were rated as “seen.” This scenario is not implausible, as it might be the case that participants with shorter RTs (subset “Fast”) consciously perceive the liminal cue stimulus less often than participants with longer RTs (subset “Slow”). For the sake of consistency, we simulated the inverse pattern for each level of balancedness: at levels 1–49, subset “Fast” was the subset with short RTs, while at levels −1 to −49 the same subset showed long RTs. In the “random” case, participants were randomly assigned to the subsets so that there was no relationship between the participants' subset membership and the pattern of subjective visibility ratings (data not shown). In both cases, 1,000 data sets were generated for each level of balancedness. Crucially, no effect of factor “visibility” was simulated, neither in the correlated case nor in the random case.

## Statistical outcomes (results)

Data analysis focused on the relative frequency of significant (*p* < 0.05) tests of the two-level factor “visibility” (i.e., false positive rate), as resulting from the 1,000 rm-ANOVAs and 1,000 LMMs at each level of balancedness. The rm-ANOVA was calculated using the aov_car() function of the *afex* package in R. Type III sums of squares were used, as these are default in many commercially available statistical packages (e.g., SPSS). The LMM was calculated using the *lmerTest* package in R, with Satterthwaite's approximation of degrees of freedom. The LMM included the two-level factor “visibility” (cond) as fixed factor as well as random slope [“RT ~ (1+cond|subj) + cond”]. Note that “subset” was not part of the LMMs, as the relationship between the participants' subset membership and the subjective visibility ratings was assumed to be unknown to the researcher prior to the experiment.

Figure 1C plots the mean RT difference between the two visibility conditions across all levels of balancedness, separately for the “correlated” case and the random case. “Mean RT” refers here to the global mean across the 1,000 mean RT differences from the simulated data sets. As expected, the mean RTs vary around zero, since no effect of factor “visibility” was simulated.

Figure 1D plots the false positive rate for the rm-ANOVA. For each level of balancedness, the false positive rate is close to 0.05. Figure 1E plots the false positive rate for LMMs. When the level of balancedness is approx. below 10, the false positive rate begins to exceed 0.05. For maximally unbalanced data, the false positive rate is about 30%. However, this increase of false positives is only observed in the correlated case.

Figure 1F plots the mean LMM estimates for the fixed effect “visibility” across all levels of balancedness. In the random case, the estimates vary around zero. In the correlated case, the estimates deviate from zero for massively unbalanced data sets (i.e., toward the left/right limits of the x-axis), in agreement with the increased false positive rate.

**Figure 2:**
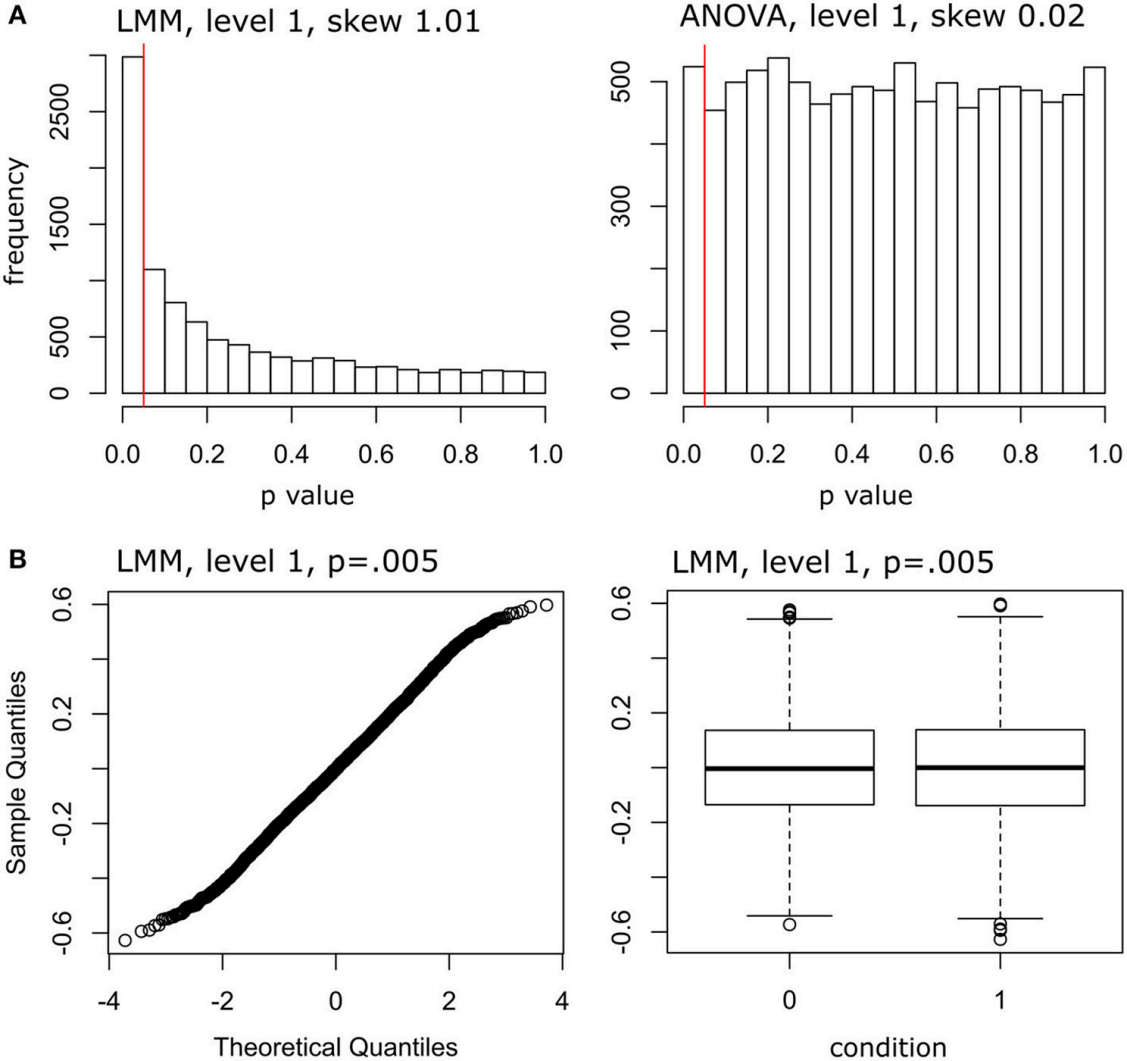
Distribution of *p*-values and diagnostic plots. **(A)** Distribution of *p*-values from 1000 LMMs. Right panel: Distribution of *p*-values from 1,000 rm-ANOVAs. The red vertical lines indicate *p* = 0.05. The skewness was computed using the skewness() function of the *moments* package in R. **(B)** Normal Q-Q plot of LMM residuals for one simulated data set with *p* = 0.005. Right panel: Boxplot of LMM residuals for one simulated data set with *p* = 0.005, separately for condition 0 (“not seen”) and condition 1 (“seen”). Data from maximally unbalanced data sets (level 1) in the correlated case.

Figure 2 provides a number of diagnostic plots. Figure 2A plots the p-value distributions of rm-ANOVA and LMM, when the data are maximally unbalanced (balancedness level 1, correlated case). As can be seen, the distribution is heavily skewed in the case of LMM. Figure 2B plots the residuals for one simulated data set with *p* = 0.005 (balancedness level 1, correlated case). The figure suggests that a violation of the normality assumption cannot explain the observed increase in false positives for LMMs.

## Conclusion

LMMs yielded strikingly more false positives than rm-ANOVA in the case of massively unbalanced within-participant data sets, when a previously unknown grouping factor remained unaccounted for. In the specific example used in this data report, the LMMs frequently indicated an effect of factor “visibility,” while only a RT difference between two subsets of participants was present in the simulated data. One solution to this problem would be to include the rating behavior of each participant in the linear model (e.g., the ratio between “seen” and “not seen” trials). The behavior of LMMs in more realistic and complex scenarios (e.g., including participants with missing data in one visibility condition) awaits further investigation. Follow-up data simulations should also investigate the rate of false negatives in similar scenarios.

While LMMs are more flexible than rm-ANOVA, and therefore are becoming increasingly popular in experimental psychology, researchers using LMMs for unbalanced data sets should be aware of the caveat described in this data report. It is generally advisable to carefully visualize the data prior to analysis, as well as to consult diagnostic plots when using LMMs. Finally, if a data set turns out to be massively unbalanced such that subsets of participants have only few trials at a specific factor level, or that participants show very different patterns of visibility ratings, the experimental design per se might have to be reconsidered.

## Author contributions

The author confirms being the sole contributor of this work and approved it for publication.

### Conflict of interest statement

The author declares that the research was conducted in the absence of any commercial or financial relationships that could be construed as a potential conflict of interest.

## Footnotes

^1^Please note that statistical test including a factor that was not under experimental control are generally considered exploratory, not confirmatory (de Groot, 2014).
